# An investigation into the role of inherited *CEACAM* gene family variants and colorectal cancer risk

**DOI:** 10.1186/s13104-022-05907-6

**Published:** 2022-02-03

**Authors:** Anna L. W. Huskey, Nancy D. Merner

**Affiliations:** 1 Department of Pathobiology, College of Veterinary Medicine, Auburn University, 1130 Wire Road, Auburn, AL 36849 USA; 2 Department of Drug Discovery and Development, Harrison School of Pharmacy, Auburn University, 3306 Walker Building, Auburn, AL 36849 USA

**Keywords:** Colorectal cancer, CEACAM, TCGA, Inherited, Familial, Genetics, Genetic risk, Risk variant

## Abstract

**Objective:**

This study was designed to determine if *CEACAM* mutations are associated with inherited risk of colorectal cancer. Recently, protein-truncating mutations in the *CEACAM* gene family were associated with inherited breast cancer risk. That discovery, along with aberrant expression of *CEACAM* genes in colorectal cancer tumors and that colorectal cancer and breast cancer share many risk factors, including genetics, inspired our team to search for inherited *CEACAM* mutations in colorectal cancer cases. Specifically utilizing The Cancer Genome Atlas (TCGA) blood-derived whole-exome sequencing data from the colorectal cancer cohort, rare protein-truncating variants and missense variants were investigated through single variant and aggregation analyses in European American and African American cases and compared to ethnic-matched controls.

**Results:**

A total of 34 and 14 different *CEACAM* variants were identified in European American and African American colorectal cancer cases, respectively. Nine missense variants were individually associated with risk, two in African Americans and seven in European Americans. No identified protein-truncating variants were associated with CRC risk in either ethnicity. Gene family and gene-specific aggregation analyses did not yield any significant results.

**Supplementary Information:**

The online version contains supplementary material available at 10.1186/s13104-022-05907-6.

## Introduction

Colorectal cancer (CRC) is the fourth most commonly diagnosed cancer in the US [1], and the lifetime risk of development is 4–5% [1, 2]. However, this risk can increase with many factors, including a family history of CRC [1]. Approximately 30% of CRC cases are familial [2, 3], and of those cases with a known genetic cause, the majority have Lynch syndrome [4]. However, up to 30% of familial cases are estimated to be genetically unsolved [5].

Attempting to discover new CRC genetic risk factors, herein, the *CEACAM* (Carcinoembryonic antigen-related cell adhesion molecule) gene family was investigated. *CEACAM* genes are a part of the Ig superfamily. These genes have diverse functions, including cell adhesion and signaling, influencing immunity, angiogenesis, and cancer [6–8]. Aberrant expression of *CEACAM* genes has long been associated with tumorigenesis, and atypical expression has been heavily linked to CRC development and progression [6, 8]. In 1965, CEA (more currently known as CEACAM5) was first identified as a tumor marker for CRC [9, 10]. Additionally, *CEACAM6* is overexpressed in CRC and has been determined to increase invasiveness [11]. Contrarily, *CEACAM1* [12, 13] and *CEACAM7* [14] have decreased expression in CRC. Furthermore, somatic mutations in *CEACAM1* [13] and *CEACAM5* [15] have been detected in CRC tumors. Nonetheless, the impact of inherited *CEACAM* gene mutations on CRC risk has yet to be determined.

Recently, rare protein-truncating variants (PTVs) in the *CEACAM* gene family were associated with the inherited risk of breast cancer [16]. That discovery, along with aberrant expression of *CEACAM* genes in CRC tumors and that CRC and breast cancer share many risk factors, including genetics [1, 17, 18], inspired our team to determine if *CEACAM* mutations are associated with CRC inherited risk.

## Main text

### Methods

Blood-derived exomes of CRC cases in The Cancer Genome Atlas (TCGA) were analyzed to investigate if *CEACAM* mutations play a role in inherited risk. Through approved research project #10805, whole-exome binary sequence alignment mapping (BAM) files were downloaded from the Genomic Data Commons (GDC) Data Portal Repository. Samples were acquired by setting specific filters. Filters under the ‘Cases’ category included Project (TCGA-COAD), Samples Sample Type (Blood-Derived Normal), and Race (‘Black or African American’ and ‘White’). The samples were further filtered under the ‘Files’ category, including Experimental Strategy (WXS) and Data Format (BAM). A total of 48 sample files were obtained for African Americans and 199 for European Americans. These files were downloaded using the GDC Data Transfer Tool (version 1.2.0).

The downloaded BAM files, which had previously been aligned to the hg38 human reference genome, were processed using the remaining portions of a pipeline adapted from the Genome Analysis Toolkit’s (GATK’s) best practices pipeline [19]. Base quality scores were recalibrated using BaseRecalibrator. Following base recalibration, the BAM files underwent coverage calculations for the exome and each *CEACAM* gene. Samtools depth function [20, 21] was used to determine the exome coverage using a BED file generated from UCSC Table Browser with the specifications: clade (Mammal), genome (Human), assembly (Dec. 2013 (GRCH38/hg38), group (Genes and Gene Predictions), track (NCBI RefSeq), and table (UCSC RefSeq (refGene)) with genome as the region of interest and “Whole Gene” selected. Samtools coverage function [20, 21] was used to generate coverage values for the *CEACAM* genes from a set of gene-specific intervals; including *CEACAM1* (NM_001184815; chr19:42507306-42528481), *CEACAM3* (NM_001815 at chr19:41796587-41811554), *CEACAM4* (NM_001817; chr19:41618971-41627074), *CEACAM5* (NM_004363; chr19:41708626-41730421), *CEACAM6* (NM_002483; chr19:41755530-41772210), *CEACAM7* (NM_006890; chr19:41673303-41688270), *CEACAM8* (NM_001816 at chr19:42580243-42594924), *CEACAM16* (NM_001039213; chr19:44699151-44710718), *CEACAM18* (NM_001278392; chr19:51478643-51490605), *CEACAM19* (NM_020219; chr19:44671452-44684355), *CEACAM20* (NM_001102597; chr19:44506159-44529675), and *CEACAM21* (NM_001098506; chr19:41576166-41586844). Furthermore, regarding variant calling, the recalibrated BAM files were converted into genome variant calling format (gVCF) files using HaplotypeCaller (GATK version 4.1.9). GenomicsDBImportant was used to generate ethnic-specific *CEACAM* gene family datasets, which were obtained by extracting the *CEACAM* gene intervals listed above. This process was followed by the GenotypeGVCFs function to generate ethnic-specific VCF files (GATK version 4.1.9). The two ethnic-specific VCF files were then annotated using ANNOVAR (version June 2020). Variants were filtered to include rare PTVs (nonsense mutations, frameshifting mutations, or splice-site affecting mutations) and missense variants with ethnic-specific minor allele frequencies (MAFs) of < 1% in Exome Variant Server (EVS; National Heart, Lung, and Blood Institute (NHLBI) Exome Sequencing Project) [22]. Each variant was individually investigated using the Fisher’s exact test [23, 24] in R (v 3.5.1), comparing MAFs of ethnic-specific TCGA CRC cases and EVS controls. Additionally, coverage values for each variant were assessed to determine the cohort’s average coverage at that genomic location. Subsequently, PTVs and missense variants were investigated together and as individual groups in gene-based and gene family-based aggregation analyses using the Fisher method through the ‘sumlog’ command as part of the ‘metap’ package within R [25, 26]. P-values were not corrected for multiple testing. Lastly, missense pathogenicity was predicted using Polyphen2 [27]. For all significant mutations, protein analysis using InterPro [28] and the Eukaryotic Linear Motif (ELM) resource [29] was carried out to identify CEACAM domains and binding motifs, respectively.

### Results

The whole-exome BAM files downloaded from TCGA had an average exome coverage of 8X, ranging from 2.3X to 21.4X among the samples. Coverage values were also generated for each *CEACAM* gene (Additional file 1: Table S1). The average coverage for the gene family was 22.9X, with 100% of the bases covered at least 1X (Additional file 1: Table S1).

After filtering for rare PTVs and missense variants in the entire *CEACAM* gene family within the TCGA CRC cohort, a total of 14 different variants were identified in African American cases (one frameshift and 13 missense; Additional file 2: Table S2), and 34 different variants were identified in European American cases (one frameshift, two splice, and 31 missense; Additional file 3: Table S3). All identified variants were heterozygous, and there were no cases of compound heterozygosity. The average coverage for the 14 variants identified in African Americans was 49X, ranging from 19 to 423X. Similarly, the average coverage for the 34 variants detected in European Americans was 42X, ranging from 3 to 923X. No identified PTVs were associated with CRC risk in either ethnicity.

In African American cases, five of the 13 missense variants were classified as probably damaging; however, none of those mutations were associated with CRC risk. Only two variants were determined to be individually associated with African American CRC risk, including *CEACAM3*:p.(Y95N) and *CEACAM8*:p.(T247A), both predicted to be likely benign (Table 1).

**Table 1:** Significant rare mutations identified in TCGA CRC African American (AA) cohort

Gene	Chr 19 position	Mutation type	Functional prediction—polyphen	cDNA change	Protein change	TCGA AA Colon MAF (%)	EVS AA MAF (%)	AA individual P-values
CEACAM3: NM_001815	41797807	missense	benign: 0.159	c.283T > A	p.(Y95N)	5.208	0.894	0.002
CEACAM8: NM_001816	42589003	missense	benign: 0.001	c.739A > G	p.(T247A)	4.167	0.931	0.015

In European American cases, 10 of the 31 missense variants were predicted to be probably damaging, but only two of which were found to be associated with CRC risk, *CEACAM1*:p.(Y68C) and *CEACAM18*:p.(C357G). A total of seven variants were determined to be individually associated with CRC in European Americans, all of which were missense variants, including the two aforementioned probably damaging missense variants and five predicted to be benign (Table 2).

**Table 2:** Significant rare mutations identified in TCGA CRC European American (EA) cohort

Gene	Chr 19 position	Mutation type	Functional prediction—polyphen	cDNA change	Protein change	TCGA EA colon MAF (%)	EVS EA MAF (%)	EA individual P-values
CEACAM1: NM_001184815	42527262	missense	probably-damaging: 1.0	c.203A > G	p.(Y68C)	0.503	0.070	0.046
CEACAM4: NM_001817	41625657	missense	benign: 0.325	c.368G > A	p.(R123E)	0.503	0.000	0.002
CEACAM8: NM_001816	42589735	missense	benign: 0.005	c.425C > T	p.(P142L)	0.503	0.012	0.006
CEACAM18: NM_001080405	51483229	missense	probably-damaging: 1.0	c.1069T > G	p.(C357G)	0.503	0.059	0.036
	51483284	missense	benign: 0.013	c.1124A > G	p.(Q375R)	0.503	0.059	0.036
CEACAM19: NM_020219	44681293	missense	benign: 0.01	c.773G > C	p.(R258T)	1.005	0.093	0.001
CEACAM20: NM_001102597	44512936	missense	benign: 0.062	c.1445C > T	p.(T482I)	0.503	0.000	0.002

Gene family and gene-specific aggregation analyses did not yield any significant results, including a combined assessment of PTVs and missense variants, as well as group analyses of PTVs, missense mutations, and probably damaging missense mutations.

### Discussion

Upon surveying the *CEACAM* gene family for rare PTVs and missense variants in CRC cases from TCGA and controls from the EVS, no gene-based or gene family-based associations with inherited risk of CRC were revealed. These results were unexpected due to the previous association of rare PTVs in the *CEACAM* gene family with inherited breast cancer risk [16], the known similarities between breast cancer and CRC risk [1, 17, 18], and the dis-regulation of *CEACAM* genes in CRC tumors [6, 8–15]. Moreover, it has been demonstrated that *CEACAM* gene function can be affected by even minor genetic changes [27], and specific residues within CEACAM proteins are crucial for normal function [12, 30, 31].

Despite the lack of association from aggregation analyses, individual variants were associated with CRC inherited risk (Tables 1 and 2). All associations involved individual missense variants; none involved PTVs, unlike the association of *CEACAM* PTVs with breast cancer risk [16]. Only four different PTVs were detected amongst all CRC cases, none of which overlapped between ethnicities. In European American CRC cases, two splice variants were detected, including *CEACAM7*:c.64 + 1G > T and *CEACAM21*:c.882 + 1G > A, and a frameshift mutation was detected, *CEACAM20:*p.(F542Sfs*56). One frameshift mutation was detected in an AA CRC case, *CEACAM21*:p.(T32Pfs*47).

**Fig. 1:**
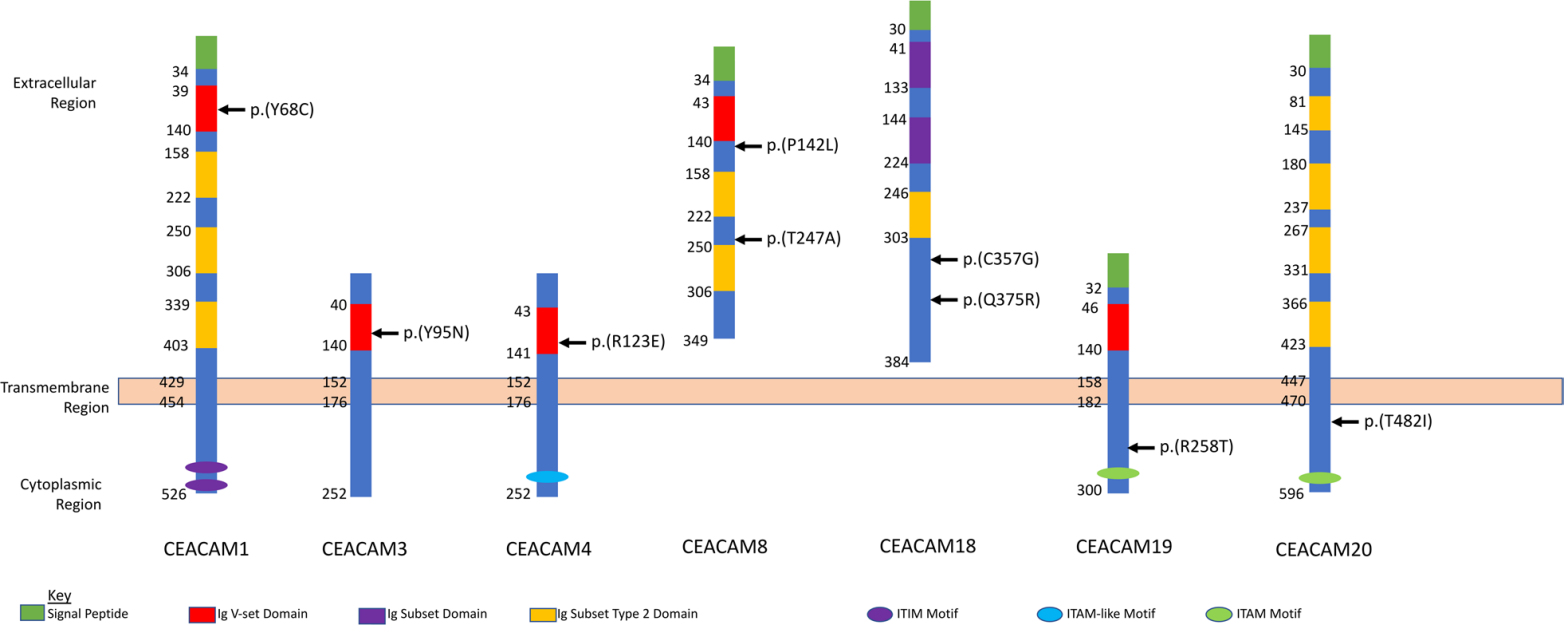
Domain analysis of the significant rare mutations identified in TCGA-COAD cohort

Overall, 9 missense variants were determined to be individually associated with risk, two in African Americans and seven in European Americans. Three associated variants were within the Ig V-set (variable) domain (Fig. 1), including *CEACAM1*:p.(Y68C) and *CEACAM4*:p.(R123E), which were associated with European American CRC risk, and *CEACAM3*:p.(Y95N), which was associated with African American CRC risk (Fig. 1). The Ig V-set domain is crucial for the dimerization of many CEACAM proteins and their ability to function within normal ranges [31, 32]. In CEACAM1, mutating particular residues within the Ig V-set domain can affect the monomer-homodimer exchange and result in the protein staying in a monomeric state [31]. CEACAM1’s ability to dimerize is required for proper function [33–36]. Knowing that CEACAM1 dimerization is crucial and CEACAM1’s current role in CRC [12, 13], *CEACAM1*:p.(Y68C) is a probable CRC inherited risk factor. *CEACAM3*:p.(Y95N) has been reported as benign in ClinVar; however, limited information was provided for that clinical classification [37]. Considering *CEACAM3* has potential links to CRC [38, 39], validating the association of *CEACAM3*:p.(Y95N) with AA CRC inherited risk is crucial in identifying possible risk factors. Lastly, *CEACAM4* has been previously associated with thyroid cancer [40], but its role in CRC is unknown. Missense variants within the Ig V-set domain identified in this study could result in repressed dimerization and require further investigation.

Two statistically significant missense variants were identified in both *CEACAM8* and *CEACAM18*. The two variants in *CEACAM8*, p.(P142L) and p.(T247A), were associated with CRC risk in European American and African American cases, respectively, and occur between functional domains of the protein (Fig. 1). Even though the role of these variants is unclear, CEACAM8 forms dimers with CEACAM6 and CEACAM1 [32, 35], both of which have previous associations with CRC [11–13]. *CEACAM18* p.(C357G) and p.(Q375R) were significantly associated in European American CRC, and p.(C357G) was predicted to be pathogenic through PolyPhen2 [27]. These mutations occur after known functional domains for CEACAM18 (Fig. 1) but could influence how the protein interacts with the cell membrane. Beyond these two *CEACAM18* variant associations, there is no known link between CEACAM18 and CRC.

A single missense mutation in both *CEACAM19* [p.(R258T)] and *CEACAM20* [p.(T482I)] was associated with European American CRC. Both of these mutations occur within the cytoplasmic region of the protein but before the ITAM binding motifs (Fig. 1). The possible impacts of these mutations are unclear; however, *CEACAM19* and *-20* have previous cancer links [41–45]. Furthermore, *CEACAM20* has been determined to play a role in gut microbiome regulation [46, 47]. The microbiome is known to influence CRC risk and progression [1], which could explain *CEACAM20’s* role in CRC risk. Additionally, *CEACAM* gene expression is altered in Inflammatory Bowel Disease (IBD)[38, 48], another well-established risk factor for CRC [49–51]. Exploring how *CEACAM* mutations and aberrant expression result in both IBD and CRC is extremely important. Unfortunately, IBD diagnoses were unavailable for TCGA CRC cases to explore that link.

Overall, this study aimed to determine if inherited *CEACAM* variants play a role in CRC risk. No gene- or gene family-based associations were identified, but nine individual missense variants in seven different *CEACAM* genes appear to be associated with inherited CRC risk. Further investigation is warranted.

## Limitations

It is important to note that the TCGA CRC cohort is not a hereditary/familial CRC cohort. Though *CEACAM* variants do not appear to play a significant role in this cohort, studying hereditary/familial CRC cohorts could reveal different findings. Such investigations are important considering that a large percentage of inherited CRC is suspected to be influenced by lower penetrant variants compounded with environmental factors [1, 5]. Furthermore, the TCGA CRC cohort was subdivided by ethnicity, and European American cases were represented ~ 4X more than African American cases. This underrepresentation is a concerning limitation, as African Americans have the highest CRC incidence and mortality rates of all ethnicities in the United States [52]. Both TCGA CRC ethnic groups had a limited number of cases, and with the prevalence of previous research linking the *CEACAM* genes to spontaneous CRC [6, 8, 11–15, 38, 39, 53–55], more genetic and functional investigations of the *CEACAM* gene family should be carried out.

## Supplementary Information

**Additional file 1: Table S1.** Coverage values for the *CEACAM* genes.

**Additional file 2: Table S2.** Full list of rare (MAF < 1%) CEACAM mutations in African American TCGA-COAD cohort and EVS African American cohort. This includes rare stop gain, frameshifting, splice-site and missense mutations identified in the “Black or African American” TCGA-COAD cohort and the EVS African American cohort.

**Additional file 3: Table S3.** Full list of rare (MAF < 1%) CEACAM mutations in European American TCGA-COAD cohort and EVS European American cohort. This includes rare stop gain, frameshifting, splice-site and missense mutations identified in the “White or Caucasian” TCGA-COAD cohort and the EVS European American cohort.

## Data Availability

The datasets supporting the conclusions of this article are available in The Cancer Genome Atlas GDC data portal TCGA-COAD repository, https://portal.gdc.cancer.gov/projects/TCGA-COAD.

## Abbreviations

AA: African American
BAM: Binary sequence alignment mapping
CRC: Colorectal cancer
ELM: Eukaryotic linear motif
EA: European American
EVS: Exome variant server
GATK’s: Genome analysis toolkit’s
gVCF: Genome variant calling format
GDC: Genomic data commons
MAFs: Minor allele frequencies
NHLBI: National Heart, Lung, and Blood Institute
PTVs: Protein-truncating variants
TCGA: The Cancer Genome Atlas
